# The Incidence of Poor Postoperative Recovery Characterized Using ‘Days Alive and Out of Hospital’ in Octogenarians and Nonagenarians—A Retrospective Cohort Study

**DOI:** 10.3390/jcm14217666

**Published:** 2025-10-29

**Authors:** Shiri Zarour, Yotam Weiss, Lisa Globerman, Michal Itkin, Sarah Saxena, Idit Matot, Barak Cohen

**Affiliations:** 1 Division of Anesthesia, Intensive Care, and Pain Management, Tel-Aviv Medical Center, Tel-Aviv University, Tel-Aviv 6423906, Israel; shiri.zarour@nhs.net (S.Z.); yotamw@tlvmc.gov.il (Y.W.); iditm@tlvmc.gov.il (I.M.); 2 Department of Peri-Operative Medicine, University College London Hospitals NHS Trust, London NW1 2BU, UK; 3 Department of Surgery, UMons Research Institute for Health Sciences and Technology, University of Mons, 7000 Mons, Belgium; sarahksaxena@gmail.com; 4 Department of Anesthesiology, Helora, 7000 Mons, Belgium; 5 Outcomes Research Consortium, Houston, TX 77030, USA

**Keywords:** older adults, elderly, postoperative outcomes, harm-benefit balance, patient-centered outcomes, risk factors, surgical futility, non-beneficial treatment

## Abstract

**Background**: Studies assessing poor postoperative recovery using patient-centered metrics among older adults are scarce. We aimed to explore poor postoperative recovery in octogenarians and nonagenarians, characterized using the validated patient-centered tool ‘days alive and out of hospital’ (DAOH). **Methods**: This retrospective cohort study included patients aged ≥ 80 years who had non-palliative surgery at a tertiary academic center between January 2017 and July 2021. We explored the incidence of DAOH at 90 days (DAOH_90_) ≤ 45 days as a pragmatic patient-centered marker of poor postoperative recovery. We also identified independent risk factors associated with this outcome using logistic regression models. Sensitivity analyses were performed using similar regression models. **Results**: Among 3683 included patients (median age 84 years), 640 patients (17%) had poor postoperative recovery. Of them, 240 patients (38%) survived the 90-day postoperative period but suffered a cumulative hospitalization period of over 45 days, and 400 patients (62%) died during the 90-day postoperative period. The most significant risk factors were ASA physical status classification (adjusted odds ratio (aOR) 3.52 [95% CI 2.55–4.87] for class 3E-5E compared to class 1–2), renal failure (aOR 3.49 [2.01–6.06] for GFR < 15 compared to GFR > 60 mL/min/1.73 m^2^), and high-risk surgery (aOR 1.85 [1.47–2.32]). **Conclusions**: We found a non-trivial rate of poor postoperative recovery in octogenarians and nonagenarians. DAOH_90_ ≤ 45 days is a simple, clear, and intuitive tool that may enhance patient-centered research and promote communication about expected outcomes, support shared decision making, and provide personalized risk assessment aligned with older patients’ goals of care.

## 1. Introduction

The number of surgical procedures performed in older adults is constantly increasing. Interestingly, this is mostly the result of the increasing rate of surgical procedures among older adults, and to a lesser extent due to the aging of the population [1,2,3]. Considering high rates of adverse postoperative outcomes among octogenarians and nonagenarians, and the increase in surgical interventions among them, concerns regarding excessive incidence of poor postoperative recovery in the geriatric population has become more pertinent [4,5].

There is a worldwide recognition that the broad spectrum of meaningful postoperative recovery in older adults is better assessed using patient-centered outcomes, including time at home, functional status, and quality of life (QOL) [6,7,8]. Nonetheless, such large population studies remain scarce, presumably due to the difficulties in utilizing qualitative measures to evaluate surgical recovery in large-scale studies [9,10].

Given the challenges of using QOL and functional status measurements in large datasets, the metric ‘days alive and out of hospital’ (DAOH) has emerged as a promising patient-centered outcome assessing the broader impact of medical interventions using administrative healthcare data [6,8,10,11,12,13]. DAOH is defined as the number of days a patient remains alive and out of the hospital within a specified time frame following an index event. This measure captures key aspects such as hospitalization duration, readmissions, and mortality, which reflects on overall life impact [11,12,13].

Poor DAOH is strongly associated with impaired functional status and diminished QOL, and is a validated surrogate for these conditions [14,15,16,17,18,19]. It reflects on older patients’ perspectives regarding goals of care priorities, such as home time, QOL, and functional autonomy, using a single objective standardized measure [20,21,22]. Thus, DAOH appears to be an appropriate tool for large-scale studies assessing the broad spectrum of poor postoperative recovery in older adults.

In this study, we sought to assess poor postoperative recovery among older adults, which will be both clinically meaningful for patients and feasible to use in large-scale cohorts. We therefore aimed to describe the incidence of poor postoperative recovery in octogenarians and nonagenarians, characterized using DAOH, and to identify risk factors associated with this outcome.

## 2. Materials and Methods

### 2.1. Study Design and Population

This single-center retrospective cohort study was approved by the institutional review board, which waived the need for individual consent. The study followed the Strengthening the Reporting of Observational Studies in Epidemiology (STROBE) reporting guidelines.

We included all patients 80 years and older presenting for non-cardiac surgery at the Tel-Aviv Medical Center from January 2017 to July 2021. In case of multiple surgeries during the study period, the first surgery within the study period was considered as the index event.

Patients were excluded if they were discharged on the same day or had missing crucial data (missing mortality data for foreign residents that are not properly reported in the National Mortality Registry). We also excluded minor surgeries as well as palliative surgeries, since we considered these procedures as potentially beneficial regardless of the associated DAOH. Minor surgery was defined as an operation magnitude classification of “minor”, in accordance with the operation severity component of the Physiological and Operative Severity Score for the Enumeration of Mortality and Morbidity (POSSUM) [23,24,25]. Palliative surgery was defined as non-curative surgery aimed at improving QOL, allowing for mobility and independence, controlling symptoms, improving vision and hearing, or relieving pain (e.g., hip fracture surgeries, spinal cord cordotomies or cingulotomies, kyphoplasties, cataract surgery, hearing aid implantation, surgical feeding jejunostomies, and proctology procedures).

### 2.2. Data Collection

Data were obtained from institutional electronic health records, including electronic medical charts, anesthesia record keeping systems, electronic operative reports, and electronic laboratory records, for the 90 days following the index surgery.

Data were retrieved using MDClone (MDClone Ltd., Be’er Sheva, Israel; version 6), a self-service data acquisition platform that enables data retrieval from electronic medical records databases (http://www.mdclone.com, accessed on 11 October 2025). To ensure data reliability, a random sample of 20% of patient records was manually reviewed, and full concordance with the extracted data was confirmed.

Data included: patient baseline characteristics, pre-existing comorbidities, chronic medications, preoperative laboratory results, surgical and anesthetic features, cumulative in-hospital length of stay (LOS) within 90 postoperative days following the index surgery, and mortality data.

### 2.3. Primary Outcome

The primary outcome was ‘days alive and out of hospital’ during the initial 90 days after the index surgery (DAOH_90_), which was based on previous data indicating that DAOH_90_ is the preferred metric to capture meaningful recovery after surgery in older comorbid patients [19].

DAOH_90_ was calculated using mortality and hospitalization data from the date of the index surgery to the 90th postoperative day, consistent with previous works [18,19]. Total in-hospital LOS was determined by adding the length of hospitalization after the index surgery to all subsequent hospital stays during the initial 90 postoperative days. DAOH_90_ was set to zero if mortality occurred during the initial 90 postoperative days, as is universally accepted (Supplementary Figure S1).

In the primary analysis, we explored the incidence of DAOH_90_ ≤ 45 days as a pragmatic patient-centered marker of poor postoperative recovery among older adults. We also identified risk factors associated with this outcome. We sought a patient-centered threshold that would be clear, intuitive, and meaningful for patients. This threshold, representing either mortality or a cumulative hospitalization time of over half of the 90-day postoperative period, aims to reflect a postoperative course with more burden than benefit in order to create a meaningful and intuitive metric for both patients and caregivers. Importantly, this choice also aligns with the findings of Spurling et al. [26], in which the lower quartile of DAOH_90_ was found to be 46 days.

Suggestions to support the primary outcome threshold using a statistically driven approach prompted a preliminary analysis, which demonstrated an 11% 90-day mortality rate and a lower 10th percentile of DAOH_90_ ≤ 51 among survivors. Applying the lower 10th percentile as a threshold on the entire study population would have resulted in the domination of mortality on the primary outcome, thereby diminishing the patient-centered focus of our study. Furthermore, the statistically driven combined composite outcome of 90-day mortality and the lower 10th percentile among survivors (DAOH_90_ ≤ 51) was highly comparable to our *a-priori* threshold of DAOH_90_ ≤ 45, but lacked the clarity and clinical interpretability required for a meaningful patient-centered outcome measure. We therefore chose to adhere to the *a-priori* threshold of DAOH_90_ ≤ 45 to provide a more intuitive and clinically meaningful patient-centered discussion.

#### Confounding Variables

The following variables were considered for potential confounding based on pre-defined clinical plausibility or association with the studied outcome in the univariate analyses (*p* < 0.1), and were therefore included in the multivariable analysis:

Baseline characteristics: age, sex, body mass index (BMI), and American Society of Anesthesiologists (ASA) physical status classification [3,4,5,27] frailty (defined as modified frailty index score ≥ 2) [28,29].

Pre-existing comorbidities and chronic medications: diabetes mellitus, ischemic heart disease, atrial fibrillation, history of cerebrovascular accident (CVA) or transient ischemic attack (TIA), chronic obstructive pulmonary disease, congestive heart failure, peripheral vascular disease, dementia, as well as chronic use of anticoagulants. All these were defined according to the ICD-9 codes listed in the pre-admission comorbidities/chronic medications sections in the electronic medical record.

Preoperative laboratory results: plasma hemoglobin concentration and the glomerular filtration rate (GFR), calculated using the Chronic Kidney Disease Epidemiology Collaboration (CKD-EPI) creatinine equation [30].

Surgical and anesthetic features: high-risk surgery (defined as classification of “major”/”major plus” in the operation severity component of the POSSUM score) [23,24,25], urgent surgery (defined according to the ASA definition of emergency surgery) [27], and surgery duration.

### 2.4. Sensitivity Analyses

Since interaction was found between the ASA physical status classification and surgical urgency, we assessed independent risk factors for the primary outcome in the following subgroups: patients with ASA physical status classification 1–2, patients with ASA physical status classification 3–5, patients having non-urgent surgeries, and patients having urgent surgeries.

### 2.5. Statistical Analyses

Categorical variables were summarized as frequency and percentage. Continuous variables were evaluated for normal distribution using histograms. Normally distributed continuous variables were reported as mean and SD, and the others were reported as median with IQR. The assumption of linearity of independent continuous variables was assessed by the Box–Tidwell test. Continuous variables that violated the assumption of linearity (BMI, plasma hemoglobin concentration, and GFR) were categorized in accordance with clinical considerations [31,32,33].

Categorical variables were compared between patients with and without the primary outcome using Chi-square tests, while continuous and ordinal variables were compared using the Independent Samples *t*-test or Mann–Whitney test. Variables that met the pre-defined significance threshold in the univariate analyses (*p* < 0.1) or were pre-defined as potential confounders based on clinical plausibility were added to the multivariable regression model.

Subgroups and interactions were examined using the Chi-squared Automatic Interaction Detection (CHAID) method. Main effects identified for interaction were combined to an interaction term, which was added to all regression models used to investigate the primary outcome. The Wald test was used to compare models with and without the interaction term. Multicollinearity between clinically related variables was assessed using the variance inflation factor (VIF). A VIF > 10 was considered indicative of multicollinearity.

Logistic regression models were used to identify independent risk factors for the studied outcome in the primary analysis as well as in the sensitivity analyses. The Hosmer–Lemeshow goodness-of-fit test was used to assess model calibration.

All statistical tests were two-sided and *p* < 0.05 was considered statistically significant. Models were considered calibrated if *p* > 0.05. SPSS software was used for all statistical analyses (IBM SPSS statistics for Windows, version 27, IBM Corporation, Armonk, NY, USA, 2020). Supplementary Figure S1 was generated using Napkin, a generative artificial intelligence tool (www.napkin.ai, accessed on 11 October 2025).

## 3. Results

**Figure 1:**
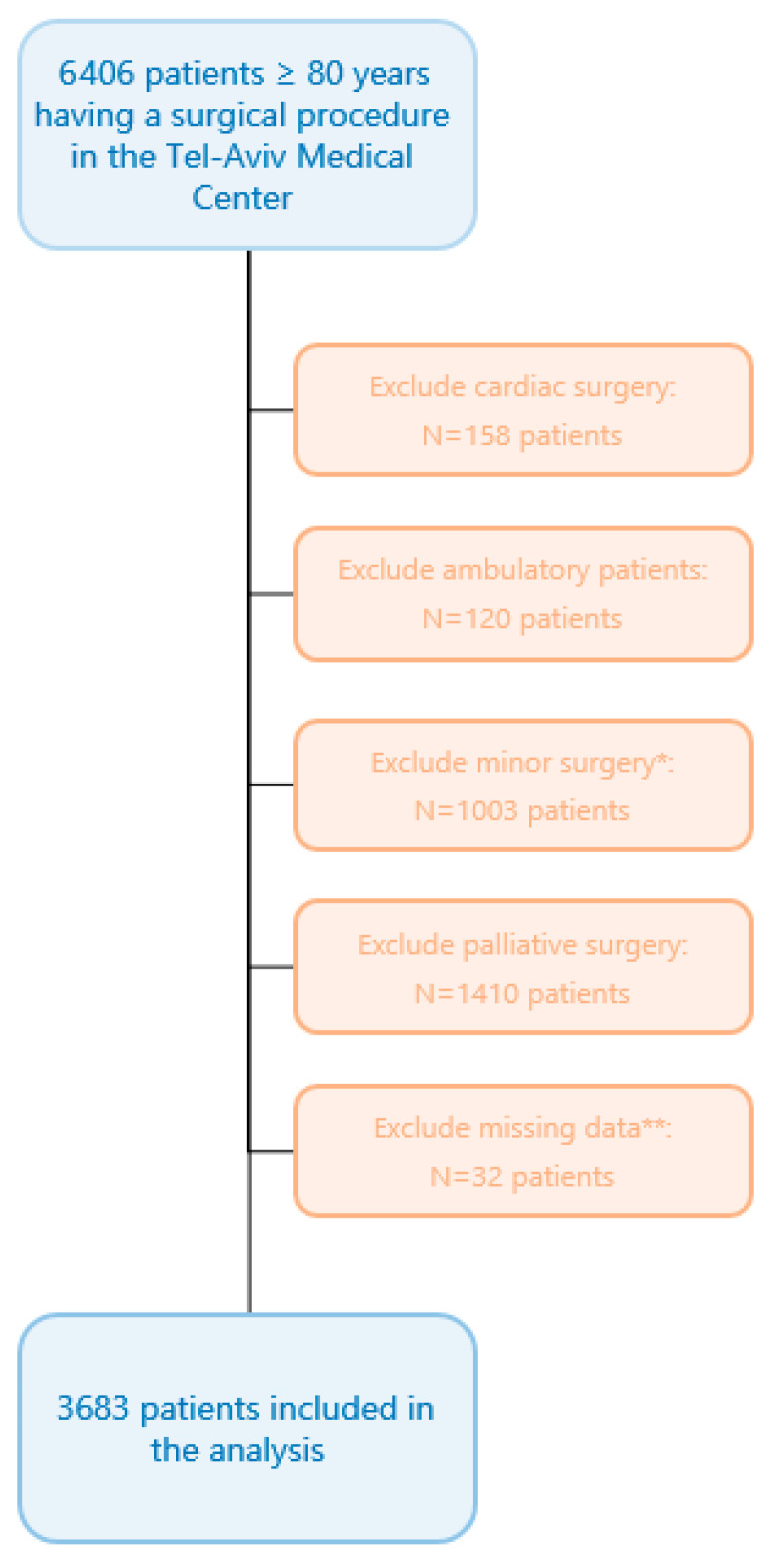
Flow chart. * Minor surgery was defined as an operation magnitude classification of “minor” in accordance with the operation severity component of the Physiological and Operative Severity Score for the Enumeration of Mortality and Morbidity (POSSUM) [23,24,25]. ** Missing crucial data were defined as missing mortality data for foreign residents not properly reported in the National Mortality Registry.

Overall, 6406 patients ≥ 80 years underwent a surgical procedure during the study period. Of these, 3683 met all enrolment criteria (Figure 1), and none were lost to follow-up. Median [IQR] age was 84 [82, 87] years, and 1930 (52%) were women. Most patients (*n* = 2434, 66%) had an ASA physical status score of 3–5. As for the features of surgical procedures, 1046 (28%) were urgent and 2123 (58%) were categorized as high-risk surgeries. Other baseline and intraoperative variables are presented in Table 1. The median [IQR] DAOH_90_ was 81 [61, 86] days. Overall, 400 patients died during the initial 90 postoperative days, with a median [IQR] time to death of 24 [10, 49] days.

### 3.1. Primary Analysis

In total, 640 patients (17%) met the primary outcome definition. Of them, 240 patients (38%) survived the 90-day postoperative period but suffered a cumulative hospitalization period of over 45 days, and 400 patients (62%) died during the initial 90 postoperative days. The univariate analysis demonstrated significant differences between patients with and without the primary outcome. As shown in Table 1, patients with DAOH_90_ ≤ 45 were older, had higher ASA scores, and higher rates of comorbidities and laboratory disturbances. In addition, they had more urgent and high-risk surgeries, as well as longer surgery durations.

**Figure 2:**
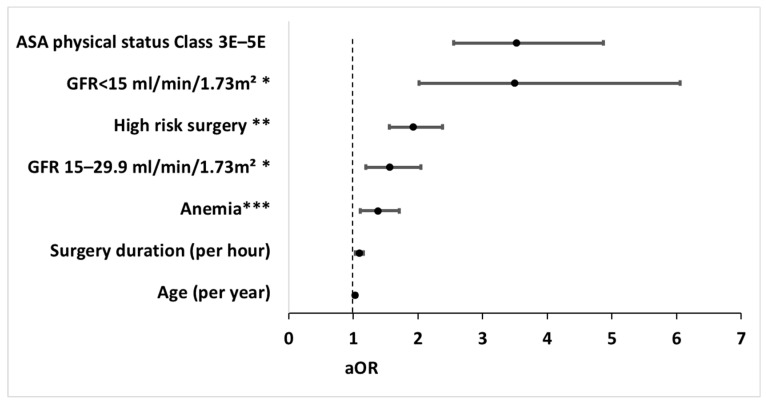
Forest plot of independent risk factors for poor postoperative recovery in the multivariable analysis. Risk factors independently associated with poor postoperative recovery, as reflected by days alive and out of hospital during the initial 90 postoperative days (DAOH_90_) ≤ 45 in the multivariable analysis. Circles represent adjusted odd ratios, while bars extend to the corresponding 95% confidence intervals. aOR, adjusted odds ratio; CI, confidence interval; ASA, American Society of Anesthesiologists; GFR, glomerular filtration rate. * GFR was categorized according to the Kidney Disease Improving Global Outcomes (KDIGO) categories for GFR [30,33]. ** High-risk surgery was defined as the operation magnitude classification of major/major+ in accordance with the operation severity component of the Physiological and Operative Severity Score for the Enumeration of Mortality and Morbidity (POSSUM) [23,24,25]. *** Anemia was defined as hemoglobin < 13.0 g/dL for males or <12.0 g/dL for females [32].

Our examination identified interaction between ASA physical status classification 3–5 and surgical urgency, which were therefore combined to an interaction term (ASA physical status classification 3E–5E) that was added to the regression models. The interaction term was independently associated with the primary outcome, while the main effects (i.e., ASA physical status classification 3–5, urgent surgery) were not (Figure 2). The regression model performed better with the interaction term than without it. Of note, no further interactions between clinically relevant variables were found, and the multicollinearity assessment was negative.

The multivariable analysis demonstrated the following independent risk factors for poor postoperative recovery: ASA physical status classification 3E–5E with adjusted odds ratio (aOR) of 3.52 [95% CI 2.55–4.87], GFR categories (aOR 3.49 [2.01–6.06] and 1.51 [1.19–2.05] for GFR < 15 and GFR < 30, respectively, compared to GFR > 60 mL/min/1.73 m^2^), high-risk surgery (aOR 1.85 [1.47–2.32]), anemia (aOR 1.38 [1.11–1.71]), surgery duration (aOR 1.09 [1.03–1.16] per hour), and age (aOR 1.03 [1.00–1.05] per year) (Figure 2). The regression model used for the primary analysis demonstrated *p* = 0.35 and was therefore considered calibrated. The multivariable regression model is presented in Supplementary Table S1.

### 3.2. Sensitivity Analyses

In accordance with the interaction found between ASA physical status classification and surgical urgency, the four subgroups demonstrated relatively unique patterns regarding independent risk factors for the primary outcome (Supplementary Tables S2 and S3).

## 4. Discussion

In this retrospective cohort study, we analyzed the data of 3683 patients 80 years or older who underwent non-cardiac, non-palliative surgical procedures. One of every six surgical procedures was followed by poor postoperative recovery, as reflected by DAOH_90_ ≤ 45 days. The most significant risk factors for poor postoperative recovery were ASA physical status classification 3E–5E, preoperative renal failure, and high-risk surgery. These findings demonstrate that poor postoperative recovery is common in octogenarians and nonagenarians, with results aligning with prior research on the significant impact of surgical urgency, pre-existing comorbidities, and surgical magnitude on postoperative outcomes in older adults [34].

Our ability to compare the current findings with existing knowledge is limited due to the scarcity of data on DAOH after various surgical procedures in older adults. However, the median [IQR] DAOH_90_ of 81 [61, 86] days observed in our cohort appears to align with previous studies, considering the differences in study population characteristics. A study of patients aged ≥ 40 years undergoing only high-risk elective surgeries reported a median [IQR] DAOH_90_ of 86 [84, 87] days, which is appropriately higher given that our population was older and had a higher rate of urgent surgeries, yet included only about 60% high-risk surgeries [18]. In addition, a significantly lower median [IQR] DAOH_90_ of 54 [0, 76] was observed in a cohort of patients aged ≥ 70 years who underwent hip fracture surgery, plausibly due to prolonged hospital stays, frequent readmissions, and the high mortality rates associated with this particularly high-risk group [35].

Nearly 40% of cases that resulted in poor postoperative recovery survived the 90-day postoperative period but suffered a cumulative hospitalization period of over half of this period (45 days). This highlights that relying solely on mortality would have missed a substantial portion of poor outcomes, particularly those most important to older adults, underscoring the importance of incorporating patient-centered metrics into postoperative evaluations. These findings might also raise important dilemmas regarding the appropriateness of certain surgical interventions in older adults with significant comorbidities. 

Maintaining home time, independence, and QOL are the most important goals of care for older adults in cases of acute diseases. Furthermore, life extension is considered least important [20,21,36,37,38]. For some patients, diminished home time, loss of independence, and reduced QOL, which have all been previously associated with poor DAOH, may be worse than the disease itself, leading to physical, emotional, and psychological burdens [14,15,16,17,39,40]. The inclusion of DAOH_90_ as a patient-centered metric aligns with these preferences, offering a more comprehensive understanding of the harm–benefit balance of surgery in this population.

Our findings have significant implications for perioperative decision making in older adults, which is crucial to ensure that suggested treatments align with their individual goals, values, and priorities [20,41,42,43,44]. For many elderly patients, maintaining home time, autonomy, and QOL may outweigh the potential benefits of surgery [43]. DAOH may serve as a useful tool to inform older patients regarding the risk–benefit ratio of surgery in a way that aligns better with their goals of care, with DAOH_90_ ≤ 45 days as a meaningful, clear, and intuitive threshold which can help provide a clearer picture of the likely outcomes, thus enabling patients to make decisions that are more informed.

It is important to recognize that the appropriateness of any medical intervention should be considered on an individual basis, taking into account each person’s goals, values, and priorities. Nonetheless, objective, clear, and relevant information should be provided to support this inherently subjective decision-making process. While characterizing poor postoperative recovery using a binary threshold may appear overly simplistic, it is a necessary step to enable risk stratification based on a well-defined outcome that is both objective and patient-centered, and to facilitate future large-scale, international, multicenter studies. Ultimately, this approach can help generate crucial high-quality, evidence-based, patient-centered data, which remain limited to this day.

Our study has several unique strengths. To our knowledge, this study is the first to describe DAOH after various types of surgeries, yet specifically among older adults. The use of a large and diverse cohort improves the study’s generalizability. It is also among the first to utilize DAOH to characterize poor postoperative recovery, emphasizing the importance of using patient-centered outcomes, which extends beyond the narrow perspective of postoperative survival when evaluating surgical outcomes. Finally, excluding palliative and ambulatory surgeries focused the analysis on interventions where postoperative recovery is a critical consideration.

Our analysis has several important limitations. First, this is a single-center study, limiting the generalizability of our findings to other healthcare settings. Second, DAOH_90_ calculations were restricted to readmissions within the study hospital, potentially underestimating poor outcomes among patients treated at other facilities or in non-hospital settings such as nursing homes. Finally, while DAOH_90_ is a promising patient-centered metric, further research is needed to validate older patients’ perspectives regarding its appropriateness as an outcome measure for non-beneficence.

## 5. Conclusions

In conclusion, we found a non-trivial rate of poor postoperative recovery among older surgical patients, underscoring the significant potential burden of prolonged hospitalizations, frequent readmissions, and diminished functional status and QOL. All of these might outweigh the potential benefits of surgical interventions in this vulnerable population. Future research should focus on validating DAOH_90_ ≤ 45 days as an appropriate measure of non-beneficence from the perspectives of older patients. If established as such, DAOH_90_ ≤ 45 could serve as a basis for developing risk stratification tools, aimed at predicting poor recovery and surgical futility, that align with older patients’ preferences. These tools could, in turn, help perioperative caregivers counsel patients and families about the benefits and risks of surgery that matter most to them, thereby enhancing shared decision making in elderly surgical populations.

## Figures and Tables

**Table 1:** Demographic, medical, surgical, and laboratory variables of patients according to postoperative outcome. Values are presented as numbers (proportion) or median [IQR].

	Missing Values	Patients with DAOH_90_ ≤ 45 (*n* = 640)	Patients with DAOH_90_ > 45 (*n* = 3043)	*p*-Value
Age (years)		86 [83, 89]	84 [82, 87]	<0.001
Sex (female)		340 (53%)	1590 (52%)	0.687
BMI category 1	195			
Underweight		28 (5%)	81 (3%)	0.021
Normal weight		289 (49%)	1314 (45%)	
Overweight		190 (32%)	1030 (36%)	
Obese		86 (15%)	470 (16%)	
ASA physical status score 3–5	15	542 [85%]	1892 [62%]	<0.001
Surgery duration (min)	13	160 [105, 239]	148 [101, 213]	<0.001
Urgent surgery 2		350 (55%)	695 (23%)	<0.001
High-risk surgery 3	11	463 (72%)	1660 (55%)	<0.001
Frailty 4		398 (62%)	1461 (48%)	<0.001
Hypertension		515 (81%)	2445 (80%)	0.994
Diabetes mellitus		226 (35%)	902 (30%)	<0.001
Malignancy		222 (35%)	1139 (37%)	0.191
Ischemic heart disease		208 (33%)	813 (27%)	<0.001
Atrial fibrillation		174 (27%)	538 (18%)	<0.001
History of CVA/TIA		117 (18%)	341 (11%)	<0.001
Chronic obstructive pulmonary disease		93 (15%)	337 (11%)	0.013
Congestive heart failure		97 (15%)	222 (7%)	<0.001
Peripheral vascular disease		59 (9%)	147 (5%)	<0.001
Dementia		50 (8%)	162 (5%)	0.014
Anticoagulants		165 (26%)	526 (17%)	<0.001
Insulin		45 (7%)	148 (5%)	0.025
Steroids		33 (5%)	119 (4%)	0.150
Anemia 5	36	415 (66%)	1341 (45%)	<0.001
GFR category 6	724			
GFR > 60 mL/min/1.73 m^2^		274 (45%)	1349 (58%)	
GFR = 30–59.9 mL/min/1.73 m^2^		152 (25%)	624 (27%)	<0.001
GFR = 15–29.9 mL/min/1.73 m^2^		153 (25%)	332 (14%)	
GFR < 15 mL/min/1.73 m^2^		36 (6%)	39 (2%)	

Data are presented as *n* (%) or median [IQR] as appropriate. Chi-square tests were used to compare prevalence rates between the groups. DAOH, days alive and out of hospital; BMI, body mass index; ASA, American Society of Anesthesiologists; CVA, cerebrovascular accident; TIA, transient ischemic attack; GFR, glomerular filtration rate. 1 BMI was categorized according to the Centers for Disease Control’s BMI categories [31]. 2 Urgent surgery was defined according to the ASA definition of emergency surgery [27]. 3 High-risk surgery was defined as the operation magnitude classification of major/major+ in accordance with the operation severity component of the Physiological and Operative Severity Score for the Enumeration of Mortality and Morbidity (POSSUM) [23,24,25]. 4 Frailty was defined as a modified frailty index score ≥ 2 [28,29]. 5 Anemia was defined as hemoglobin < 13.0 g/dL for males or <12.0 g/dL for females [32]. 6 GFR was categorized according to the Kidney Disease Improving Global Outcomes (KDIGO) categories for GFR [30,33].

## Data Availability

The data that support the findings of this study are available from the corresponding author upon reasonable request.

## Supplementary Materials

The following supporting information can be downloaded at: https://www.mdpi.com/article/10.3390/jcm14217666/s1, Figure S1. Calculating DAOH90 in postoperative care. Days alive and out of hospital (DAOH). Table S1. Multivariable regression model used for the primary analysis. Table S2. Results of the urgent and non-urgent subgroup analyses compared to the primary analysis. Table S3. Results of the ASA physical status class 1–2 and class 3–5 subgroup analyses compared to the primary analysis.

## Abbreviations

The following abbreviations are used in this manuscript:

DAOH	Days alive and out of hospital
DAOH90	Days alive and out of hospital at 90 days
QOL	Quality of life
STROBE	The Strengthening the Reporting of Observational Studies in Epidemiology
POSSUM	The Physiological and Operative Severity Score for the Enumeration of Mortality and Morbidity
LOS	Length of stay
BMI	Body mass index
ASA	American Society of Anesthesiologists
CVA	Cerebrovascular accident
TIA	Transient ischemic attack
ICD	International Classification of Disease
GFR	Glomerular filtration rate
CKD-EPI	The Chronic Kidney Disease Epidemiology Collaboration
SD	Standard deviation
IQR	Interquartile range
CHAID	Chi-squared Automatic Interaction Detection
