# Supplementary material for: The Incidence of Poor Postoperative Recovery Characterized Using ‘Days Alive and Out of Hospital’ in Octogenarians and Nonagenarians—A Retrospective Cohort Study
Source: J Clin Med. 2025 Oct 29;14(21):7666. doi: 10.3390/jcm14217666 (PMC12609303; doi:10.3390/jcm14217666)
Supplement: Supplementary file 1 [file jcm-14-07666-s001.zip › jcm-3901722-supplementary.pdf]

Supplementary table 1: Multivariable regression model used for the primary analysis

Covariate	P-value	aOR	95% CI
ASA physical status (compared to class 1-2)			
Class 1E-2E	0.535	1.190	0.687 - 2.062
Class 3-5	0.091	1.320	0.957 - 1.821
Class 3E-5E	0.000	3.520	2.547 - 4.865
GFR category[1] (compered to GFR>60 ml/min/1.73m²)			
GFR = 30-59.9 ml/min/1.73m²	0.634	1.061	0.831 - 1.356
GFR = 15-29.9 ml/min/1.73m²	0.001	1.561	1.190 - 2.048
GFR<15 ml/min/1.73m²	0.000	3.490	2.011 - 6.058
High-risk surgery[2]	0.000	1.846	1.468 - 2.321
Anemia[3]	0.004	1.378	1.111 - 1.710
Surgery duration (per hour)	0.004	1.090	1.028 - 1.155
Age (per year)	0.032	1.027	1.002 - 1.052

Congestive heart failure	0.056	1.375	0.992 - 1.906
Frailty[4]	0.051	1.298	0.999 - 1.687
History of CVA/TIA	0.060	1.295	0.989 - 1.695
Ischemic heart disease	0.186	0.856	0.679 - 1.078
Chronic obstructive pulmonary disease	0.310	1.166	0.866 - 1.570
Anticoagulants	0.317	1.167	0.862 - 1.580
Dementia	0.321	1.208	0.832 - 1.754
Atrial Fibrillation	0.372	1.147	0.848 - 1.552
BMI category[5] (compared to normal weight category)			
Underweight	0.512	1.180	0.720 - 1.935
Overweight	0.647	0.948	0.754 - 1.191
Obese	0.422	0.885	0.656 - 1.193
Diabetic mellitus	0.719	0.955	0.745 - 1.226
Peripheral vascular disease	0.777	1.055	0.729 - 1.526

aOR, adjusted odds ratio; CI, confidence interval; ASA, American Society of Anesthesiologists; GFR, glomerular filtration rate; CVA, Cerebrovascular Accident; TIA, Transient Ischemic Attack; BMI, Body mass index.

1 GFR was categorized according to the Kidney Disease Improving Global Outcomes (KDIGO) categories for GFR. [30,33]

2 High-risk surgery was defined as operation magnitude classification of major/major+ in accordance with the operation severity component of the Physiological and Operative Severity Score for the Enumeration of Mortality and Morbidity (POSSUM).[23-25]

3 Anemia was defined as hemoglobin <13.0 g/dL for males or <12.0 g/dL for females. [32]

4 Frailty was defined as modified frailty index score≥2. [28,29]

5 BMI was categorized according to the Centers for Disease Control's BMI categories.[31]

Supplementary Table 2: Results of the Urgent and Non-urgent subgroup analyses compared to the primary analysis

	Primary analysis			Non-urgent surgeries subgroup			Urgent surgeries subgroup		
**Covariate**	**P-value**	**aOR**	**95% CI**	**P-value**	**aOR**	**95% CI**	**P-value**	**aOR**	**95% CI**
**ASA physical status (compared to class 1-2)**									
**Class 3E-5E**	<0.001	3.52	2.55 - 4.87	N/A	N/A	N/A	N/A	N/A	N/A
**Class 3-5**	0.091	1.32	0.96 - 1.82	0.078	1.35	0.97 - 1.89	<0.001	2.82	1.67 - 4.77
**GFR category[1] (compered to GFR>60 ml/min/1.73m²)**									
**GFR<15 ml/min/1.73m²**	<0.001	3.49	2.01 - 6.06	0.157	2.00	0.77 - 5.25	<0.001	4.76	2.29 - 9.89
**GFR = 15-29.9 ml/min/1.73m²**	0.001	1.56	1.19 - 2.05	0.053	1.49	0.10 - 2.22	0.013	1.62	1.11 - 2.37
**GFR = 30-59.9 ml/min/1.73m²**	0.634	1.06	0.83 - 1.36	0.494	1.12	0.81 - 1.54	0.825	0.96	0.65 - 1.41
**High risk surgery[2]**	<0.001	1.85	1.47 - 2.32	<0.001	1.73	1.27 - 2.36	<0.001	1.99	1.40 - 2.83
**Anemia[3]**	0.004	1.38	1.11 - 1.71	0.010	1.48	1.10 - 1.99	0.104	1.31	0.95 - 1.80
**Age (per year)**	0.032	1.03	1.00 - 1.05	0.422	1.02	0.98 - 1.05	0.049	1.04	1.00 - 1.07
**Surgery duration (per hour)**	0.004	1.09	1.03 - 1.16	0.224	1.05	0.97 - 1.13	0.003	1.16	1.05 - 1.28
**Congestive heart failure**	0.056	1.38	0.99 - 1.91	0.046	1.60	1.01 - 2.52	0.397	1.23	0.77 - 1.96
**History of CVA/TIA**	0.060	1.30	0.99 - 1.70	0.682	1.09	0.73 - 1.62	0.013	1.63	1.11 - 2.39
**Atrial Fibrillation**	0.372	1.15	0.85- 1.55	0.509	1.15	0.76 - 1.73	0.567	1.14	0.73 - 1.80
**Frailty[4]**	0.051	1.30	0.99 - 1.69	0.115	1.34	0.93 - 1.93	0.224	1.27	0.87 - 1.86
**Dementia**	0.321	1.21	0.83 - 1.75	0.831	0.93	0.49 - 1.78	0.181	1.39	0.86 - 2.23
Peripheral vascular disease	0.777	1.06	0.73 - 1.53	0.083	1.57	0.94 - 2.60	0.283	0.75	0.44 - 1.27
**Anticoagulants**	0.317	1.17	0.86 - 1.58	0.194	1.31	0.87 - 1.96	0.936	1.02	0.64 - 1.62

**Chronic obstructive pulmonary disease**	0.310	1.17	0.87 - 1.57	0.460	1.17	0.78 - 1.75	0.501	1.17	0.75 - 1.82
**Diabetes Mellitus**	0.719	0.96	0.75 - 1.23	0.389	0.86	0.61 - 1.22	0.631	1.09	0.76 - 1.58
**Ischemic heart disease**	0.186	0.86	0.68 - 1.08	0.025	0.69	0.50 - 0.95	0.745	1.06	0.75 - 1.49
**BMI category[5] (compared to normal weight category)**									
**Underweight**	0.512	1.180	0.720 - 1.935	0.791	1.11	0.51 – 2.42	0.568	1.21	0.63 - 2.33
**Overweight**	0.647	0.948	0.754 - 1.191	0.416	0.88	0.64 – 1.20	0.938	1.01	0.72 - 1.43
**Obese**	0.422	0.885	0.656 - 1.193	0.833	1.96	0.64 – 1.43	0.231	0.76	0.48 - 1.20

aOR, adjusted odds ratio; CI, confidence interval; ASA, American Society of Anesthesiologists; GFR, glomerular filtration rate; CVA, Cerebrovascular Accident; TIA, Transient Ischemic Attack; BMI, Body mass index.

1 GFR was categorized according to the Kidney Disease Improving Global Outcomes (KDIGO) categories for GFR. [30,33]

2 High-risk surgery was defined as operation magnitude classification of major/major+ in accordance with the operation severity component of the Physiological and Operative Severity Score for the Enumeration of Mortality and Morbidity (POSSUM).[23-25]

3 Anemia was defined as hemoglobin <13.0 g/dL for males or <12.0 g/dL for females. [32]

4 Frailty was defined as modified frailty index score≥2. [28,29]

5 BMI was categorized according to the Centers for Disease Control's BMI categories.[31]

Supplementary Table 3: Results of the ASA physical status class 1-2 and ASA physical status class 3-5 subgroup analyses compared to the primary analysis

	**Primary analisys**			**ASA physical status score 1-2 subgroup**			**ASA physical status score 3-5 subgroup**		
	**P-value**	**aOR**	**95% CI**	**P-value**	**aOR**	**95% CI**	**P-value**	**aOR**	**95% CI**
**ASA physical status (compared to class 1-2)**									
**Class 3E-5E**	<0.001	3.52	2.55 - 4.87	N/A	N/A	N/A	N/A	N/A	N/A
**Class 1E-2E**	0.091	1.32	0.96 - 1.82	N/A	N/A	N/A	N/A	N/A	N/A
**URGENT SURGERY**	N/A	N/A	N/A	0.884	1.045	0.58 - 1.88	<0.001	2.76	2.19 - 3.48
**GFR category[1] (compered to GFR>60 ml/min/1.73m²)**									
**GFR<15 ml/min/1.73m²**	<0.001	3.49	2.01 - 6.06	0.254	4.47	0.34 - 5.59	<0.001	3.41	1.93 - 6.04
**GFR = 15-29.9 ml/min/1.73m²**	0.001	1.56	1.19 - 2.05	0.006	2.80	1.35 - 5.83	0.015	1.44	1.07 - 1.93
**GFR = 30-59.9 ml/min/1.73m²**	0.634	1.06	0.83 - 1.36	0.713	1.11	0.63 - 2.00	0.900	1.02	0.77 - 1.34
**High risk surgery[2]**	<0.001	1.85	1.47 - 2.32	0.175	1.44	0.85 - 2.44	<0.001	1.94	1.50 - 2.51
**Anemia[3]**	0.004	1.38	1.11 - 1.71	0.587	1.15	0.70 - 1.90	0.004	1.43	1.12 - 1.82
**Age (per year)**	0.032	1.03	1.00 - 1.05	0.000	1.12	1.00 - 1.19	0.610	1.01	0.98 - 1.04
**Surgery duration (per hour)**	0.004	1.09	1.03 - 1.16	0.119	1.12	0.971 - 1.30	0.011	1.09	1.02 - 1.16
**Congestive heart failure**	0.056	1.38	0.99 - 1.91	0.574	0.56	0.08 - 4.17	0.033	1.44	1.03 - 2.02
**History of CVA/TIA**	0.060	1.30	0.99 - 1.70	0.895	1.06	0.42 - 2.68	0.039	1.35	1.02 - 1.79
**Atrial Fibrillation**	0.372	1.15	0.85- 1.55	0.969	0.98	0.37 - 2.61	0.289	1.19	0.86 - 1.64
**Frailty[4]**	0.051	1.30	0.99 - 1.69	0.251	0.65	0.31 - 1.36	0.963	1.01	0.77 - 1.32
**Dementia**	0.321	1.21	0.83 - 1.75	0.137	1.59	0.86 - 2.94	0.141	1.25	0.93 - 1.67
Peripheral vascular disease	0.777	1.06	0.73 - 1.53	0.130	1.88	0.83 - 4.27	0.638	1.08	0.79 - 1.49
**Anticoagulants**	0.317	1.17	0.86 - 1.58	0.543	1.34	0.53 - 3.39	0.385	1.15	0.84 - 1.59

**Chronic obstructive pulmonary disease**	0.310	1.17	0.87 - 1.57	0.254	2.31	0.55 - 9.75	0.961	0.99	0.68 - 1.45
**Diabetes Mellitus**	0.719	0.96	0.75 - 1.23	0.661	0.76	0.23 - 2.57	0.254	1.26	0.85 - 1.88
**Ischemic heart disease**	0.186	0.86	0.68 - 1.08	0.926	1.04	0.49 - 2.17	0.154	0.84	0.67 - 1.07
**BMI category[5] (compared to normal weight category)**									
**Underweight**	0.512	1.180	0.720 - 1.935	0.271	1.96	0.59 – 6.52	0.713	1.11	0.65 – 1.90
**Overweight**	0.647	0.948	0.754 - 1.191	0.703	0.90	0.52 – 1.55	0.785	0.97	0.75 – 1.25
**Obese**	0.422	0.885	0.656 - 1.193	0.680	1.17	0.56 – 2.41	0.287	0.84	0.60 – 1.16

aOR, adjusted odds ratio; CI, confidence interval; ASA, American Society of Anesthesiologists; GFR, glomerular filtration rate; CVA, Cerebrovascular Accident; TIA, Transient Ischemic Attack; BMI, Body mass index.

1 GFR was categorized according to the Kidney Disease Improving Global Outcomes (KDIGO) categories for GFR. [30,33]

2 High-risk surgery was defined as operation magnitude classification of major/major+ in accordance with the operation severity component of the Physiological and Operative Severity Score for the Enumeration of Mortality and Morbidity (POSSUM).[23-25]

3 Anemia was defined as hemoglobin <13.0 g/dL for males or <12.0 g/dL for females. [32]

4 Frailty was defined as modified frailty index score≥2. [28,29]

5 BMI was categorized according to the Centers for Disease Control's BMI categories.[31]

Supplementary Figure 1: Calculating $DAOH_{90}$ in postoperative care

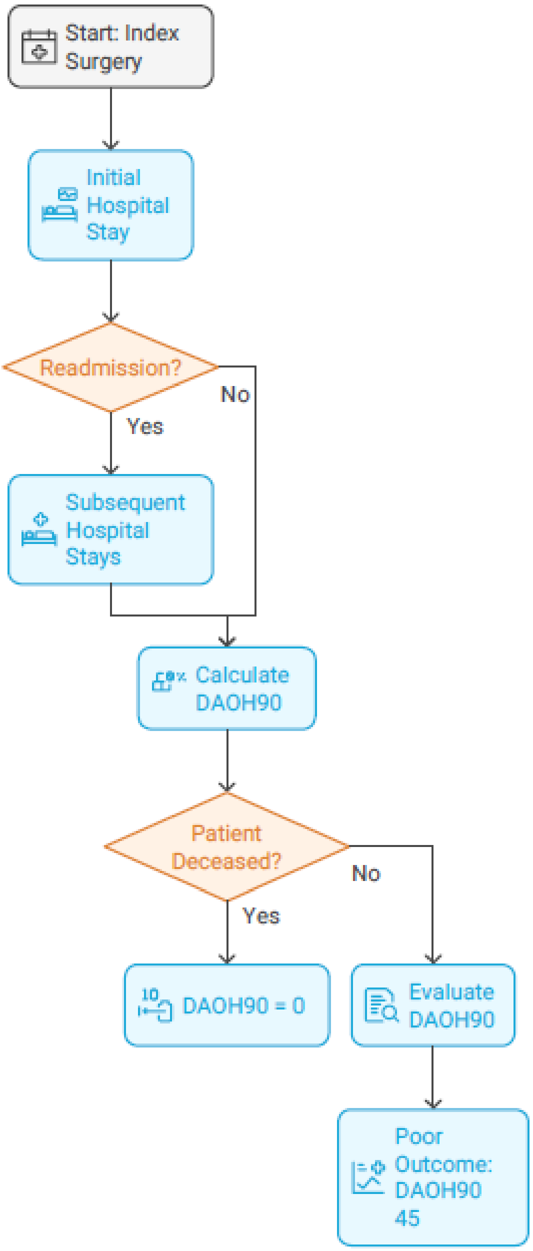

Days alive and out of hospital, DAOH.
